# A Novel *In Vitro* Model for Cancer Stem Cell Culture Using Ectopically Expressed *Piwil2* Stable Cell Line

**Published:** 2013-08-24

**Authors:** Maryam Shahali, Maryam Kabir-Salmani, Karim Nayernia, Hamid Reza Soleimanpour-Lichaei, Mohammad Vasei, Seyed Javad Mowla, Ehsan Ranaie, Mehdi Shakibaie, Mohammad Hossein Modaresi

**Affiliations:** 1 Department of Molecular Genetics, Faculty of Biological Sciences, Tarbiat Modares University, Tehran, Iran; 2 Department of Medical Biotechnology, Stem Cell Division, National Institute of Genetic Engineering and Biotechnology, Tehran, Iran; 3 Geneocell Ideal Science-Based Company, Tehran, Iran; 4 Department of Pathology, Faculty of Medicine, Shariati Hospital, Tehran University of Medicine Science, Tehran, Iran; 5 Department of Medical Genetics, Faculty of Medicine, Tehran University of Medicine Science, Tehran, Iran

**Keywords:** Cancer, Stem cells, *Piwil2*, Self-Renewal, Ectopic

## Abstract

**Objective:**

*Piwil2*, a member of Ago/Piwi gene family containing Piwi and PAZ domains, has been shown to be ectopically expressed in different cancer cells, especially its remarkable expression in cancer stem cells (CSCs), and is also known to be essential for germ line stem cell self-renewal in various organisms. The hypothesis that CSC may hold the key to the central problem of clinical oncology and tumor relapse leads to more anticancer treatment studies. Due to emerging controversies and extreme difficulties in studying of CSC, like the cells using *in vivo* models, more attempts have expended to establish different* in vitro* models. However, the progress was slow owing to the problems associated with establishing proper CSC cultures* in vitro*. To overcome these difficulties, we prompted to establish a novel stable cell line over-expressing *Piwil2* to develop a potential proper* in vitro* CSC model.

**Materials and Methods:**

In this experimental study, mouse embryonic fibroblasts (MEFs) were isolated and electroporated with a construct containing *Piwil2* cDNA under the control of the cytomegalovirus promoter (CMV). Stable transfectants were selected, and the established MEF-Piwil2 cell line was characterized and designated as CSC-like cells using molecular markers. Functional assays, including proliferation, migration, and invasion assays were performed using characterized CSC like cells in serum-free medium. Additionally, MEF-Piwil2 cell density and viability were measured by direct and indirect methods in normoxic and hypoxic conditions.

**Results:**

The results of reverse transcriptase-polymerase chain reaction (RT-PCR), western blot, and immunocytochemistry revealed an overexpression for *Piwil2* in the transfected *Piwil2* cells both in the RNA and protein levels. Furthermore, analysis of the kinetic and stoichiometric parameters demonstrated that the specific growth rate and the yield of lactate per glucose were significantly higher in the MEF-Piwil2 group compared to the MEF cells (ANOVA, p< 0.05). Also, analysis of functional assays including migration and invasion assays demonstrated a significantly higher number of migrated and invaded cells in the MEF-Piwil2 compared to that of the MEF cells (ANOVA, p< 0.05). The MEF-Piwil2 cells tolerated hypoxia mimetic conditions (CoCl_2_ ) with more than 95% viability.

**Conclusion:**

According to the molecular and functional studies, it has been realized that *Piwil2* plays a key role(s) in tumor initiation, progression and metastasis. Therefore, *Piwil2* can be used not only as a common biomarker for tumor, but also as a target for the development of new anticancer drug. Finally, the main outcome of our study was the establishment of a novel CSC-like* in vitro* model which is expected to be utilized in understanding the complex roles played by CSC in tumor maintenance, metastasis, therapy resistance or cancer relapse.

## Introduction

Cancer is defined as a diverse range of diseases in which dysregulated tissue clones grow rapidly in an uncontrolled manner and spread throughout the surrounding tissues. Several investigations have highlighted that cancer is indeed a complex multistep process requiring multiple genetic and epigenetic alterations in the expression of oncogenes, tumor-suppressor genes, cell adhesion molecules, DNA repair genes, genetic instability, telomerase activation, and cell-cycle regulators in which the accumulation of mutations in the genes directly control cancerous cell birth or death (1, 2). Among the heterogeneous populations of cancerous cells, only a tiny subset termed as cancer stem cells (CSCs) has the capacity to produce phenotypic heterogeneity in new tumors (3). In general, these cells are defined by two features: i. self-renewal and ii. muti-potency of differentiation (4). It is obvious that although the CSC hypothesis may not be true for all tumor types (5, 6), at least in hematopoetic (7) and some solid tumors (8), including pancreatic (9), colon (10), breast (11), lung (12), prostate (13), and brain (14) tumors, a small population of cells can be isolated in order to self-renew and form well differentiated tumors similar to that of the patient’s tumor from which they arise.

To consider CSCs as one of therapeutic target candidates for cancer, further studies are needed to address complexities and challenges involving their biological functions, biomarkers, signal pathways, differentiation regulation, genomics, proteomics, and CSC-specific molecules in order to isolate and/or target CSCs with high accuracy. Due to the extreme difficulties in studying CSC-like cells through *in vivo* models, more attempts were made to develop different* in vitro* model systems. However, despite the intensive efforts invested into the establishment of a proper model, to the best of our knowledge, all hitherto introduced systems encountered serious problems. Thus, one of the main targets of this study was to establish a novel* in vitro* model for CSCs that can be used as a way to better understand the molecular and cellular aspects of tumor development, progression and invasion, therapy resistance, and of course, developing new anticancer drugs.

Recent studies have indicated ectopic expression of stem cell protein *Piwil2*, a member of Ago/*Piwi* gene family containing *Piwi* and *PAZ* domains, in several cancer cells with its predominant expression in CSCs (4, 15-19). With this knowledge, we selected *Piwil2* as a causative factor for generation of CSC-like* in vitro* model to come up with the objectives of this search.

## Materials and Methods

### Isolation and culture of mouse embryonic fibroblasts (MEF)

In this experimental study, for MEF isolation, uteri isolated from 12.5-day-pregnant mice (strain NMRI from Pasteur Institute, Iran) were washed with phosphate-buffered saline (PBS). The head and liver tissues were removed from isolated embryos. The remaining bodies were washed in fresh PBS, minced and transferred into a 0.25% trypsin/1 mM EDTA solution (0.5 ml per embryo), and then, incubated at 37˚C for 10 minutes. After trypsinization, an equal amount of medium [dulbecco’s modified eagle’s medium (DMEM) containing 10% fetal bovine serum (FBS)] was added, followed by pipetting up and down a few times to help with tissue dissociation. Cells were collected by centrifugation (1000×g for 5 minutes) and cultured on 100 mm dishes in DMEM (Gibco, Uk) containing 10% FBS (Gibco, Uk), 50 units/50 mg/ ml penicillin/streptomycin (Gibco, Uk) at 37˚C with 5% CO_2_ , as previously described (20). 

### Transfection

MEFs (in passage 3) were electroporated with a plasmid carrying an empty vector (pcDNA3) or encoding *Piwil2* (pCDNA3-*Piwil2*) (15) and pCDNA3 by Neon™ Transfection System (Invitrogen, USA) according to the manufacturer’s protocol (pulse voltage: 1, 350V, pulse width: 30 ms, pulse number: 1, cell density: 5×10^6^ cells/ml, and tip type: 10 μl). Stable MEFs transfectants were selected by cultivating in medium containing 600 µg/ml of G418 (geneticin; Gibco, Uk) for 4 weeks. This established cell line was designated as MEF-Piwil2.

### RNA extraction and RT-PCR

Total RNA was extracted from MEFs and MEF*Piwil2* cells using RNA Isolation Kit (Roche, Germany) according to the manufacturer’s instructions. cDNAs were prepared from 1.5 µg RNA using M-MuLV Reverse Transcriptase and random primer (Fermentas, USA), then each PCR amplification was performed with Taq DNA Polymerase (Cinnagen, Iran). *Β2M* (beta-2 macroglobulin) housekeeping gene was used as the control (standard) gene. Primer sequences are listed in table 1.

**Table 1:** List of primer sequences used in qRT-PCR


Name	Forward	Reverse

**β2M**	CTGACCGGCCTGTATGCTAT	TTTCCCGTTCTTCAGCATTT
**piwil2**	TTGGCCTCAAGCTCCTAGAC	CATGCCACGGAACATGGAC


### Immunocytochemistry

Cells were grown on chamber slid in a tow-well plate and washed three time with PBS then fixed in 4% PFA in PBS and permeabilized with 0.5% Triton X-100 (MERCK; Germany) in PBS for 5 minutes. cells were blocked with 5% donkey serum in PBS for 1 hour at room temperature. Chamber slides were incubated with rabbit polyclonal antihuman piwil2 (16) antibody at 1:200 dilution for O/N at 4˚C and then washed with PBS and incubated for 1 hour with fluorescein-conjugated secondary antibody and DAPI (4’, 6-diamidino-2-phenylindole; Jackson; USA) at 1:200 dilutions and 1:1000, respectively. Cells were further washed in PBS and mounted with vectashield mounting medium and analyzed using fluorescence microscopy (BX51 Olympus Microscope, Korea).

### Western blot

To examine the expression of *Piwil2* at the protein level in the transfected cells, standard western blot analysis was performed. Cells were lysed in lysis buffer (CelLyticTM M cell lysis reagent, Sigma, USA), and then, total protein contents were determined by the Bradford method. Proteins (40 µg) were separated by sodium dodecyl sulfatepolyacrylamide gel electrophoresis (SDS-PAGE) under reducing conditions and transferred to a polyvinylidene difluoride membrane (PVDF; Millipore, USA). Membrane was probed with specific antibodies. Blot was washed and probed with respective secondary peroxidase-conjugated antibodies, and the bands were visualized by enhanced chemoluminescence (ECL; Najmbiotech, Iran). The following antibodies were used: rabbit polyclonal anti-human piwil2 (16) and mouse monoclonal anti- β-actin (ab8226) (as a loading control). Primary and secondary antibodies were used at 1:1000 and 1: 2000 dilutions, respectively.

### Induction of hypoxia using cobalt chloride 

1×10^5^ cells from *Piwil2*-transfected and MEF cells were plated in 6 cm cell culture dishes in DMEM medium containing 10% FBS. Cells were treated with various concentrations [0 (normoxic), 25, 50, 100, 150 and 200 µM] of CoCl_2_ (19) (Sigma-Aldrich, USA) for 48 hours. After cells were trypsinized, they were counted with a hemacytometer. Also, cell viability was quantified.

### Sample analysis 

Cell viability was determined by the Trypan blue exclusion method. Both grids of a neubauer haemocytometer slide were loaded with the cell suspension, while microscopic cell counts were performed on four large squares of each grid. Glucose and lactate concentrations were enzymatically measured (21) by glucose and lactate assay kits (ChemEnzyme Co., Iran) according to manufacturer’s protocol.

### Statistical analysis 

One-way ANOVA was used to compare the means of the obtained data. Values of p< 0.05 were considered significant. All statistical calculations were carried out with the SPSS version 16 software.

### Invasion assay 

Cell invasion assays were performed using BD BioCoat™ Matrigel™ Invasion Chambers (MIC) (Cat # 354480, 24 well format) consisting of a BD Falcon™ TC Companion Plate with Falcon Cell Culture Inserts containing an 8 micron pore size PET membrane with a thin layer of MATRIGEL Basement Membrane Matrix. Briefly, 2.5×10^4^ cells/400 µl of DMEM supplemented with 0.01% insulin transferin selenium (ITS; Gibco, UK) was plated in the upper chambers and cultured for 24 hours. DMEM with 10% FBS served as a chemoattractant in lower chambers. Cells from the upper surface of the Matrigel layer were removed by gentle swabbing, while transmigrated cells attached to the membrane were fixed in 4% paraformaldehyde and stained with eosin. The filters were rinsed with water, excised from the inserts, and mounted upside-down onto glass slides. Cells, occupying the underside of the filters and the pores, were counted in non-overlapping fields of the whole membranes under a light microscope (Zeiss, Germany). Cell invasion was tested in triplicate wells, on three independent occasions. 

### Cell wounding and migration assays 

MEFs and MEF-Piwil2 Cells were plated at 1.2-1.4×10^4^ /cm^2^ in DMEM containing 10% FBS and allowed to grow to confluence. Wounding was performed using a sterile razor blade to scrape cells off the culture plate, leaving a denuded area and a sharp visible demarcation line at the wound edge. The wounded monolayers were rinsed three times with serum-free medium and were inspected immediately after wounding. Then, for quantifying migration, sections of the wounds were selected according to the criteria described previously (22), marked, and counted. The areas with technical problems such as extra lines made by razor blade, incomplete scraping or deep wounds were not selected as one of the experimental group. The cells in both experimental groups were incubated for 36 hours. Then, cells were rinsed with PBS, fixed, and stained using Diff-Quick kit solutions (International reagents Corp, Kobe, Japan), and examined under phasecontrast microscopy (Zeiss, Germany). Migrated cells were counted in sections 500 µm in length, allowing a 20 µm space from the demarcation line in order to minimize the possible physical effects of cell movement resulting from cell proliferation. Statistical analysis was calculated by averaging a mean of 10 sections per test substance for each experiment. The number of migrated cells was expressed as mean ± SEM. Statistical significance was evaluated using analysis of variance with Scheffé’s test and p< 0.05 was considered statistically significant. The experiments were repeated at least four times in each group (MEF and MEF-Piwil2) to assess reproducibility. 

## Results

### MEF-Piwil2 generates CSC-like cells

**Fig 1:**
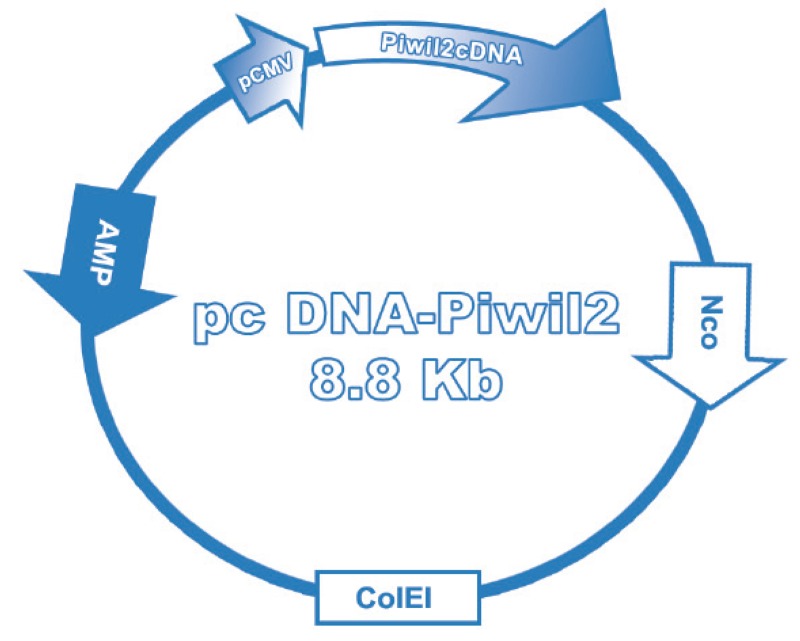
Schematic pcDNA-*Piwil2* map used for transfection of MEFs.

To establish a stable cell line over-expressing *Piwil2* for developing an* in vitro* model for CSC culture, we transfected the MEFs designated as MEF-Piwil2. The cells over-expressed mili (*Piwil2*) using a construct containing pCMV-*Piwil2*cDNA shown in figure 1.

**Fig 2:**
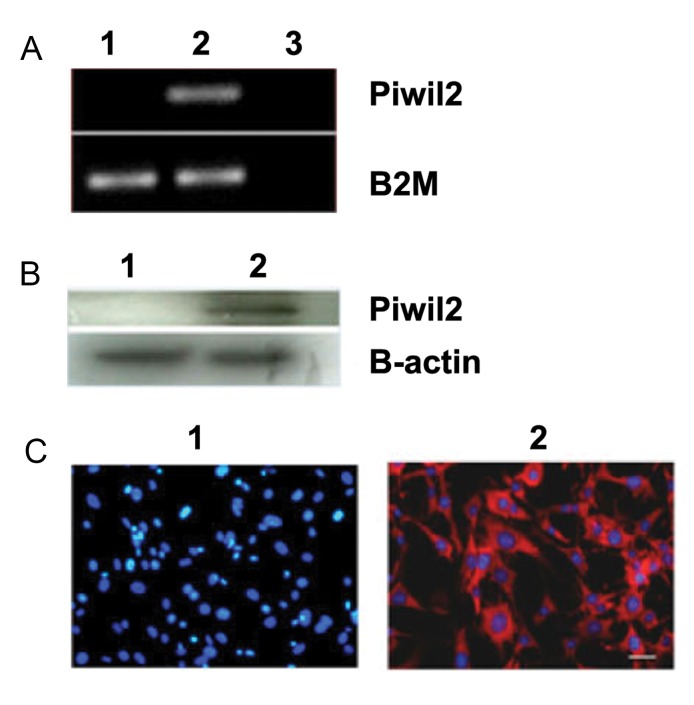
*Piwil2* expression in MEF and MEF-Piwil2 using RT-PCR, Western Blot and Immunocytochemistry methods. A. RT-PCR (normalized to B2M) analysis showed *Piwil2* expression in MEF-Piwil2 cells, while no band was observed for MEFs. B. Western-blot (β-actin was used as loading control), and C. Immunocytochemistry of both confirmed the results obtained by RT-PCR. 1. MEFs, 2. MEF-Piwil2, and 3. H2O.

The expression of *Piwil2* in the transfected stable cell line at the RNA and protein levels was confirmed by RT-PCR, western blot, and ICC (immunecytochemistry). Figure 2, panels A-C, exhibited the expression of *Piwil2* in the MEF-Piwil2 stable cell line, while the MEF cells showed no expression.

### Measurement of the kinetic and stoichiometric parameters

The kinetic and stoichiometric parameters are shown in table 2. The results showed that the yield of lactate per glucose (Y lac/glc) increased in MEF-Piwil2 cells more than 10% (ANOVA, p< 0.05) in comparison with MEF cells culture. The specific growth rate of cancer cells reached 0.036 1/h. This amount of specific growth rate was 25% higher than MEF cells 0.025 1/h. The (Y _lac/glc_) increased about 11% in high level of CoCl_2_ (200µM) compared to the control culture (MEF-Piwil2 in normoxic conditions) (ANOVA, p< 0.05)).

**Table 2:** The kinetic and stoichiometric parameters of MEF-Piwil2 and MEFs


Name	MEF cells	MEF-Piwil2 cells

**µ (1/h)**	0.025	0.036


Specific cell growth rate (µ) and the yield (lac/glu) of MEF and MEF-Piwil2 cells were measured. The growth rate of cells is defined by dx/dt=µx , where the constant " µ" (1/h) is the specific growth rate, " x" is cell density (cells/ml) and " t" is culture time (hour).

**Fig 3:**
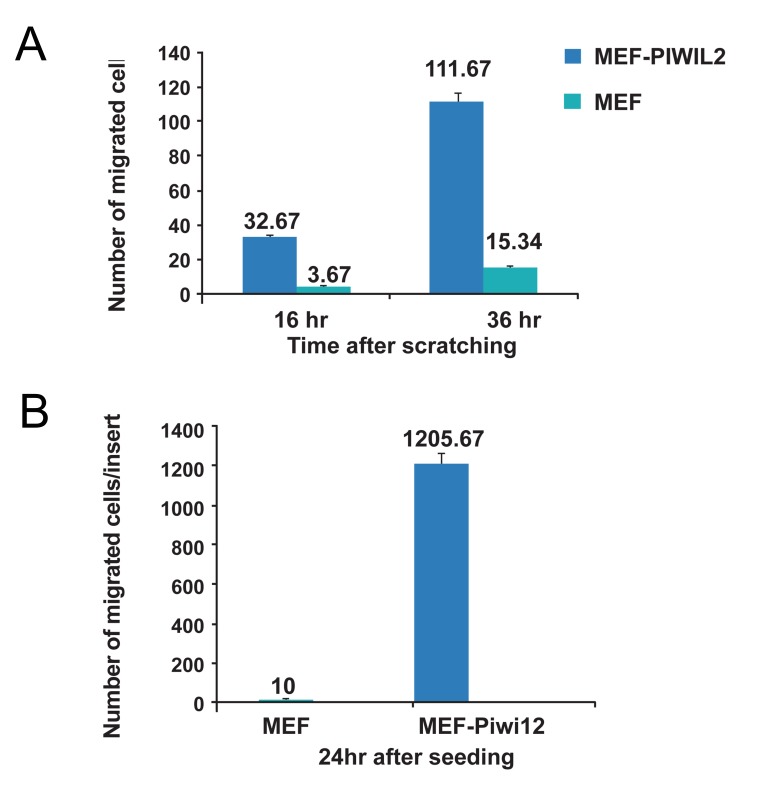
Migration (A) and invasion (B) assays in MEF-Piwil2 and MEFs (p< 0.05). A. Migration in MEF-Piwil2 cells demonstrates a significant difference compared to that of MEFs (111.67 ± 16.07 vs. to 15.34 ± 6.65 for MEF-Piwil2 and MEFs, respectively). B. Cell invasion indicated a considerable increase in MEF-Piwil2 (1205.67 ± 242.5 vs. 10 ± 6.9 for MEF-Piwil2 and MEFs, respectively).

Migratory and invasive behaviors were compared between MEF-Piwil2 and MEF cells. As shown in (Fig 3, panels A and B), both migration and invasion were dramatically increased in MEF*Piwil2* cells compared with MEFs.

### Effect of hypoxia condition to cell density of MEF-Piwil2

**Fig 4:**
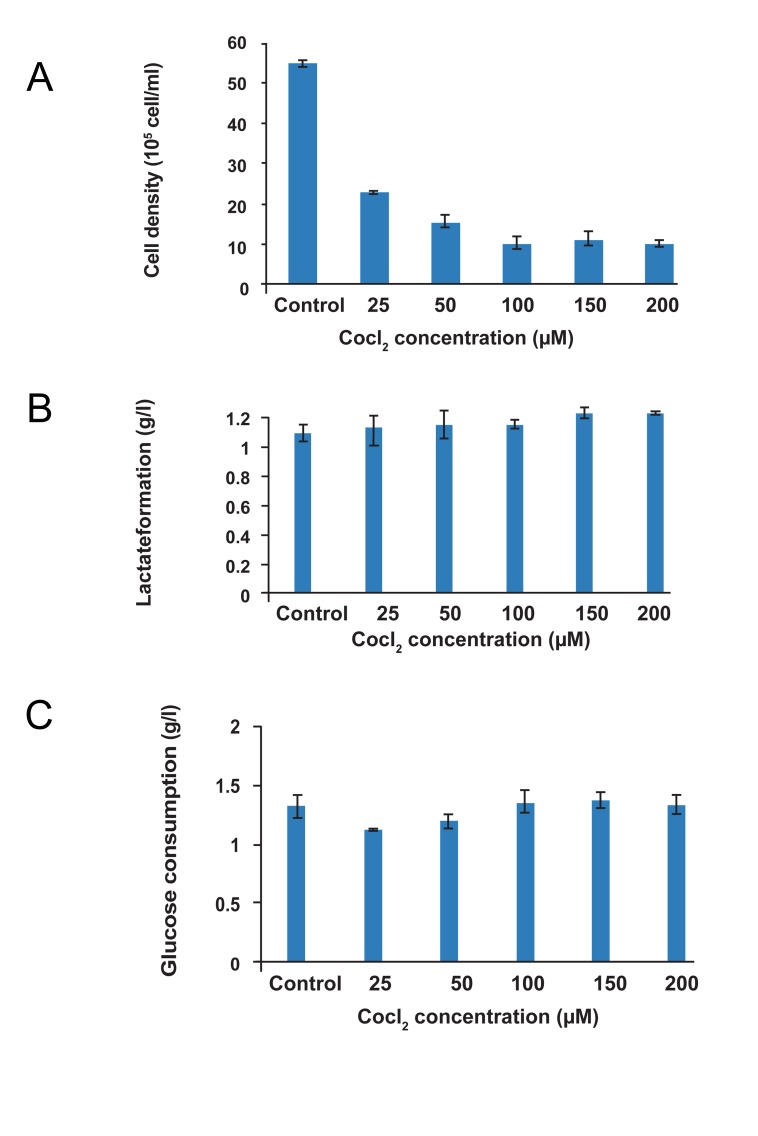
Cell density (A), lactate formation (B), and glucose consumption (C) following hypoxic exposure. Cell growing, glucose consumption, and lactate production in normoxic and hypoxic (25, 50, 100, and 200 µM CoCl_2_ ) conditions. As shown in the panel (A), the number of cells (growing cells) decreased as results of cobalt augmentation. As shown in the panel (B), glucose consumption did not increase CoCl_2_ concentration; however, cell growing decreased (1.09 ± 0.06 for normoxic condition vs. 1.23 ± 0.007 for hypoxic conditions). In panel (C), lactate formation elevated with increasing the hypoxic condition (1.32 ± 0.115 for normoxic condition vs. 1.33 ± 0.089 for hypoxic conditions). Control (MEF-Piwil2 in normoxic condition). In hypoxic condition after 8 hours, MEF cells were observed to float; therefore, further analysis was not performed.

The cell density decreased about 58, 72, and 81% in different concentrations of CoCl_2_ (25, 50, and 200 µM, respectively) compared with the cells in the control group (MEF-Piwil2 in normoxic condition), (ANOVA, p< 0.05) (Fig 4A). However, the viability remained unchanged after 48 hours of hypoxic conditions (more than 95%). 

### Effect of oxygen limitation on MEF-Piwil2 metabolism

Glucose consumption as an indirect method of cell activity was measured for all experiments. The similar glucose concentration was measured for all experiments (about 1.2 g/l). High level of lactate concentration and lower cell density MEF*Piwil2* in hypoxia mimetic conditions (different concentrations of CoCl_2_ ) in comparison with control (normoxic) condition proved the effect of DO (Dissolved Oxygen is the amount of oxygen concentration in solution) on cell energy supplementation. Lactate concentration reached 1.238 g/l and 1.09 g/l at 200 µM CoCl_2_ and control culture, respectively, after 48 hours of cultivation (Fig 4, panels B, C).

In oxygen limiting environment (anaerobic metabolism), 2 moles of lactate are produced per mol of glucose. The pyruvate resulting from the glucolysis is oxidatively decarbonised to acetyl-CoA and transformed through the Tricarboxylic acid (TCA)-cycle into water and CO_2_ . This pathway results in 30 mol ATP per mol glucose. Moreover, the lactate formation increased as another cell growth inhibitor. Based on previous papers, the lactate concentration has an inhibitory effect at concentrations of 18 mM (23).

## Discussion

CSC research owes its importance to the need for understanding the cell signaling pathways, which maintains, inflammation, epithelial to mesenchymal transition (EMT), hypoxia and angiogenesis; however, these homeostatic processes require further study to expand our understanding of cancer biology and to identify potential therapeutic targets in personalized medicine (24).

Previous studies demonstrated multiple biological functions for different members of Piwi family, like *Piwil2*. These functions include activities in germ line stem cell (GSC) self-renewal, cell Cycling, RNA interference (RNAi), chromosomal remodeling and epigenetic modulation in various organisms. Moreover, *Piwil2* activates pathways is related to apoptosis (Stat3/Bcl-XL) and proliferation (Stat3/cyclinD1) (4, 15-19, 25). According to findings, this protein was ectopically expressed in various developing stages of different cancers, predominantly in precancerous stem cells (PCSCs) (4, 17) and CSCs (16). However, it is not clear whether *Piwil2* has a role(s) in development of CSC populations or it is consequence of cancer. Thus, in the present study, the MEF-Piwil2 cell line was established, while the effect of *Piwil2* expression on its molecular and functional behavior was investigated. MEF cells were selected as useful primary cell platform in this research because usage of commercially-available cell lines could confound our results according to the following factors: random mutations and their innate inclination to cancerous state (26).

Our results confirmed proliferative and anti-apoptotic roles for *Piwil2* (increased expression of *PCNA, Stat3, Bcl-X_L_* , and *cyclinD1*), as previously reported (4, 15-17, 25). Moreover, the resulting cell line stably expressing piwil2 demonstrated a higher expression for CD44 and CD133, whereas a lower expression for CD24, which all are considered as cancer stem cell biomarkers. Our molecular data were consistent with *Piwil2* reprogramming role in the emergence or prevalence of cancer stem cell population (paper in press).

The micro-environmental physiology of tumor is characterized by higher lactate, extracellular acidosis, low oxygen gradients (hypoxia and anoxia), and glucose deprivation (27). The analysis of the kinetic and stoichiometric parameters (Table 2) indicated that in normoxic conditions, (medium without CoCl_2_ ) the specific growth rate of MEF-Piwil2 cells reached 0.036 1/h, which was 25% higher than MEF cells growth rate (ANOVA, p< 0.05). Moreover, it was found that production of MEF-Piwil2 cells lactate was considerably increased in aerobic conditions. Conversion of glucose to lactic acid in the presence of oxygen is known as aerobic glycolysis or the 'Warburg effect'. Increased aerobic glycolysis has been observed in cancerous cells (28).

acterized by their poorly vascularized regions (29); therefore, the ability of malignant cells for surviving in hypoxic conditions is of critical importance. There is increasing evidence showing a link between the ability of CSCs surviving under hypoxic conditions and its key role in therapeutic resistance (30). Interestingly, the CSC-like cells in this culture model are capable of surviving in the hypoxic conditions induced by adding cobalt chloride to the culture media. In the hypoxia mimetic cobalt chloride conditions, the yield of lactate per glucose increased about 11% in elevated levels of CoCl_2_ (200µM), which was significantly higher compared to that of the control MEF-Piwil2 cells in normoxic conditions (ANOVA, p<0.05).

After 48 hours of hypoxia, the viability of MEF*Piwil2* cells remained unchanged (more than 95%), suggesting that they were resistant to hypoxic conditions, a property that makes them a potential candidate for assessing CSC behavior under hypoxic conditions.

Functional assays including migration and invasion assays were also performed under serumfree conditions as described above. The results of statistical analysis demonstrated a significantly higher number of migrated and invaded cells in the MEF-Piwil2 compared to that of MEF cells (ANOVA, p<0.05). The cells in the MEF-Piwil2 group could survive in the serum-free conditions. Culture in this medium has been shown to cause greater chromosomal stability* in vitro* than in serum-containing medium (31).

The* in vitro* findings are in accordance with our *in vivo* observations, which it indicates that MEF*Piwil2* cells form primary and metastatic tumor rapidly within first few weeks after their transplantation into Nude mice, while MEF cells show no tumor after approximately one year in press).

Collectively, considering all molecular and functional data, it is believed that MEF-Piwil2 cells can be more efficient for research due to their potential to survive under serum-free conditions. In other words, the cell line stably expressing *Piwil2* could assume behavior similar to CSCs in terms of their potential to survive, expand, migrate and invade in the hypoxia and serum-free conditions which mimics the niche of CSCs.

## Conclusion

Our molecular and functional studies, demonstrated *Piwil2* as a key player in the process of tumor initiation, progression and invasion, suggest that this factor can be used as a common cancer biomarker, as well as a target for the development of new anticancer drug. Eventually, the major outcome of this research was the generation of a novel CSC-like* in vitro* model with remarkable application in understanding of the complex roles played by CSC in tumor main-tenance, metastasis, therapy resistance or cancer relapse.
